# Preoperative Serum Transient Receptor Potential Ankyrin 1 (TRPA1) Levels in Class III Obesity: A Prospective Controlled Observational Study

**DOI:** 10.3390/ijms27198919

**Published:** 2026-10-08

**Authors:** Nizamettin Kutluer, Onur Ağ, Tamer Gündoğdu, Mehmet Zeki Öğüt, Denizhan Kılıç, Hakan Ayyıldız, Serhat Doğan, Mehmet Buğra Bozan

**Affiliations:** 1 Department of General Surgery, University of Health Sciences, Elazığ Fethi Sekin City Hospital, Elazığ 23300, Türkiye; 2 Department of General Surgery, Elazığ Fethi Sekin City Hospital, Elazığ 23300, Türkiye; 3 Department of General Surgery, Malatya Training and Research Hospital, Malatya 44000, Türkiye; 4 Department of Biochemistry, Elazığ Fethi Sekin City Hospital, Elazığ 23300, Türkiye; 5 Department of General Surgery, Special Kayseri Dünyam Hospital, Kayseri 38010, Türkiye; drserhatdogan@gmail.com

**Keywords:** TRPA1, class III obesity, bariatric surgery, circulating biomarker, oxidative stress, metabolic inflammation, sleeve gastrectomy, ELISA

## Abstract

Transient Receptor Potential Ankyrin 1 (TRPA1) is a membrane cation channel activated by oxidative stress products that are chronically elevated in class III obesity. Although tissue-level TRPA1 biology is increasingly well described, the biological meaning of circulating immunoreactive TRPA1 remains poorly established. This single-centre, prospective, controlled observational study compared preoperative serum TRPA1 between adults with class III obesity scheduled for laparoscopic sleeve gastrectomy and contemporaneous controls, and explored metabolic and inflammatory correlates of the circulating signal. Serum TRPA1 was quantified by enzyme-linked immunosorbent assay (ELISA) in 42 consecutive surgical candidates and 36 controls. The primary analysis used continuous (log-transformed) TRPA1 with age- and sex-adjusted logistic regression; ROC-derived dichotomisation was retained only as an exploratory analysis. Serum TRPA1 did not differ between groups (median 309.8 vs. 340.5 pg/mL; Mann–Whitney U, *p* = 0.323; Hodges–Lehmann median difference [obesity − control] −30.8 pg/mL, bootstrap 95% CI −227.43 to 43.02). After adjustment for age and sex, continuous log-TRPA1 was not independently associated with obesity status (OR per log-unit 0.88, 95% CI 0.46–1.69; *p* = 0.699), and discrimination was poor (AUC 0.435; 95% CI 0.305–0.562). Established inflammatory and metabolic markers differed between groups, but serum TRPA1 did not track them. In this cohort, preoperative serum TRPA1 did not distinguish class III obesity from controls. The negative circulating finding should be interpreted as lack of evidence for a large between-group difference in ELISA-measured serum TRPA1—and as a potential translational disconnect from tissue TRPA1 biology—rather than as the definitive exclusion of any tissue-level role for the channel.

## 1. Introduction

Obesity has reached pandemic proportions worldwide and continues to rise across both high- and low-income populations [1,2]. It imposes a substantial health burden, contributing to a wide range of cardiovascular, metabolic, musculoskeletal, and psychosocial disorders that impair quality of life and shorten life expectancy. Class III obesity, conventionally defined as a BMI ≥40 kg/m^2^, represents the highest category within the traditional BMI-based classification and is commonly associated with pathological adipose tissue expansion and sustained low-grade systemic inflammation [1,2].

This inflammation is driven by macrophage infiltration of adipose tissue, dysregulated adipokine secretion, and overproduction of reactive oxygen species (ROS) and electrophilic lipid peroxidation products, including 4-hydroxynonenal (4-HNE), malondialdehyde (MDA), and acrolein [1,3,4]. Through endoplasmic reticulum stress, NF-κB-mediated cytokine production, and mitochondrial dysfunction, these mediators promote insulin resistance and major obesity-related comorbidities (type 2 diabetes mellitus, hypertension, dyslipidaemia, and cardiovascular disease). Adipose tissue inflammation is now regarded as a central biological link between obesity and metabolic dysfunction [1,2].

The Transient Receptor Potential Ankyrin 1 (TRPA1) channel is a polymodal, non-selective cation channel of the TRP superfamily [5,6]. Originally characterised in unmyelinated primary afferent neurons as a sensor of environmental irritants (allyl isothiocyanate, acrolein, and cinnamaldehyde), it is now recognised to be expressed in numerous non-neuronal tissues including macrophages, mast cells, the gastrointestinal epithelium, and adipose tissue [5,6,7,8,9]. Crucially, its principal endogenous agonists—4-HNE, hydrogen peroxide, prostaglandin J2 metabolites, and endocannabinoids—are precisely the oxidative and inflammatory mediators chronically elevated in obesity. TRPA1 activation promotes calcium influx, neurogenic inflammation, neutrophil recruitment, and cytokine release (IL-1β, IL-6, and TNF-α) [6,7,9,10], and pharmacological or dietary engagement of TRPA1-related pathways has been linked to adipose energy balance regulation, thermogenesis, and improved metabolic phenotypes in experimental models [11,12,13,14,15,16,17]. Importantly, channel activation is not synonymous with increased channel protein expression: agonist exposure may gate existing channels, desensitise them, or secondarily alter transcription depending on cell type, duration, and context [5,6,9]. As these participants are also bariatric surgery candidates, TRPA1—positioned at the interface of oxidative stress and innate inflammation—may participate in the metabolic dysregulation of class III obesity and in the inflammatory resolution that follows bariatric surgery [18,19]. Most available evidence therefore concerns tissue-level expression or functional activation rather than a validated circulating biomarker.

How a transmembrane ion channel becomes detectable in serum remains incompletely resolved. ELISA-measured circulating immunoreactive TRPA1 is likely to represent a heterogeneous mixture of ectodomain shedding, extracellular vesicles/exosomes, cellular debris, and, potentially, incompletely characterised soluble fragments rather than intact functional channels at the plasma membrane [5,9,20]. Haemolysis, leukocyte activation, or metabolic stress-related cell injury could also contribute to the measured signal and should be considered when interpreting between-group comparisons [20,21]. A small number of human studies have quantified serum TRPA1 by ELISA in non-obesity settings (for example, cognitive–olfactory research) [22], and other clinical work has examined TRPA1 on circulating leukocytes or TRPA1 gene methylation in blood rather than serum protein [21,23]; nonetheless, circulating TRPA1 remains far less established than tissue TRPA1 biology. Antibody-based detection of human TRPA1 is itself non-trivial, and commercial reagents require cautious interpretation [20]. No published clinical study has prospectively measured circulating serum TRPA1 in people with class III obesity undergoing bariatric surgery and compared it with controls. We therefore conducted a prospective, controlled observational study with two exploratory aims: (i) to determine whether preoperative serum TRPA1 concentrations differ between people with class III obesity scheduled for sleeve gastrectomy and contemporaneous controls; and (ii) to explore correlations between circulating TRPA1 and key metabolic and inflammatory indices (body mass index (BMI), homeostasis model assessment of insulin resistance (HOMA-IR), C-reactive protein (CRP), fasting glucose, and insulin). We reasoned that if chronic endogenous agonist load translated into a higher circulating immunoreactive TRPA1 signal, serum concentrations might be elevated in the obesity group; however, given the uncertain relationship between tissue activation and circulating protein, this was treated as a hypothesis to be tested rather than a mechanistic certainty. The study was not designed to re-diagnose obesity already defined by BMI, but to evaluate whether serum TRPA1 carries additional preoperative biological information in this setting.

## 2. Results

### 2.1. Participant Characteristics

A total of 78 participants were enrolled (42 with class III obesity, 36 controls; Table 1). Women comprised 31/42 (74%) of the obesity group versus 12/36 (33%) of controls (*p* < 0.001), and the obesity group was older (median 36.0 vs. 24.5 years; *p* = 0.0002; r = −0.49). All people with obesity underwent laparoscopic sleeve gastrectomy, with a median postoperative hospital stay of six days (IQR 5–7), and, as expected, had markedly higher weight and BMI. The age and sex imbalance was accounted for in the adjusted analyses.

### 2.2. Serum TRPA1

Preoperative serum TRPA1 did not differ significantly between the class III obesity group [median 309.75 pg/mL (IQR 254.15–600.35)] and controls [340.50 pg/mL (268.93–856.32)] (Mann–Whitney U, *p* = 0.323; rank-biserial r = 0.13, small effect). The Hodges–Lehmann estimated median difference (obesity − control) was −30.8 pg/mL, with a bootstrap 95% confidence interval of −227.43 to 43.02 pg/mL (2000 resamples, seed 42), indicating that the data are compatible with both a modest decrease and a modest increase and do not demonstrate the absence of all biologically conceivable differences. Fifteen measurements exceeded the manufacturer’s stated analytical range (3–900 pg/mL); these values were retained in the primary analysis as reported by the plate reader calibration curve and were not diluted and re-assayed (see Section 2.7 and Limitations). The violin plot (Figure 1) shows wide, right-skewed distributions with substantial overlap and upper-end outliers, justifying non-parametric testing and a log-scaled display.

### 2.3. Confounder-Adjusted Analysis: Logistic Regression

In the primary confounder-adjusted analysis, continuous log-transformed serum TRPA1 was entered into a binary logistic regression with obesity status as the dependent variable, together with age and sex (Table 2). After adjustment, log-TRPA1 was not independently associated with obesity status (β = −0.1285, SE = 0.3327, OR per log-unit = 0.88, 95% CI 0.46–1.69; *p* = 0.699), whereas both age (OR = 1.10 per year, 95% CI 1.04–1.17; *p* = 0.002) and male sex (OR = 0.21, 95% CI 0.07–0.62; *p* = 0.005) were independent predictors, confirming them as important confounders. The model showed adequate fit (LR χ^2^ = 26.2, *p* < 0.001; Nagelkerke R^2^ = 0.38). As an exploratory secondary approach only, serum TRPA1 was also dichotomised at the ROC-derived Youden threshold (≤524 pg/mL = low) and re-entered with age and sex. In that exploratory model, low serum TRPA1 was not independently associated with obesity status (OR = 2.30, 95% CI 0.75–7.04; *p* = 0.146). The Youden-derived cut-off was generated from the same dataset, has no external validation, and is not presented as a clinically actionable threshold.

### 2.4. Discriminatory Ability: ROC Analysis

ROC analysis assessed the exploratory ability of preoperative serum TRPA1 to discriminate people with class III obesity from controls across decision thresholds, with class III obesity as the positive class (Figure 2; Table S1). The curve tracked closely to the reference diagonal, with an area under the curve of 0.435 (bootstrap 95% CI 0.305–0.562). At the Youden-optimal threshold of 524 pg/mL, sensitivity was 73.8% and specificity 44.4% (Youden index J = 0.183). An AUC below 0.50 indicates that higher serum TRPA1 was associated with control rather than obesity status in this sample. These metrics indicate that preoperative serum TRPA1 alone does not provide useful case–control discrimination in the present cohort, consistent with the substantial distributional overlap. Any future biomarker strategy would more plausibly require multivariable integration of demographic and inflammatory covariates (for example, CRP, leukocyte indices, fasting glucose/HbA1c, and age/sex) rather than reliance on circulating TRPA1 alone.

### 2.5. Correlation Analyses

Within the obesity cohort, preoperative serum TRPA1 showed no significant correlation with BMI (rs = −0.102, *p* = 0.520, *n* = 42), HOMA-IR (rs = −0.177, *p* = 0.261, *n* = 42), fasting glucose (rs = −0.106, *p* = 0.506, *n* = 42), fasting insulin (rs = −0.188, *p* = 0.233, *n* = 42), CRP (rs = −0.289, *p* = 0.231, *n* = 19) or WBC (rs = −0.129, *p* = 0.417, *n* = 42) (Figure 3; Table S2). In controls, the corresponding associations were likewise non-significant (BMI rs = −0.140, *p* = 0.416, *n* = 36; fasting glucose rs = −0.015, *p* = 0.933, *n* = 34; CRP rs = +0.068, *p* = 0.842, *n* = 11; WBC rs = −0.231, *p* = 0.181, *n* = 35), and insulin and HOMA-IR were not analysable (*n* < 5). The weak negative association with HbA1c in the obesity group (rs = −0.314, *p* = 0.043, *n* = 42) was not reproduced in controls (rs = +0.198, *p* = 0.670, *n* = 7) and should be regarded as exploratory given multiple testing and data sparsity. These correlation patterns should not be over-interpreted as evidence that tissue TRPA1 expression is unchanged; they indicate only that the ELISA-measured circulating signal did not covary with the selected metabolic and inflammatory indices in this cross-sectional preoperative dataset. Absence of a serum–marker correlation is compatible with (i) a true lack of association for circulating immunoreactivity, (ii) limited assay dynamic range/precision, or (iii) a dissociation between circulating protein and tissue channel biology.

### 2.6. Haematological and Metabolic Outcomes

Table 3 presents the primary TRPA1 comparison alongside the haematological and biochemical outcomes. Serum TRPA1 did not differ between groups and was excluded from the multiplicity correction; Benjamini–Hochberg false discovery rate (FDR) adjustment was applied to secondary outcomes only. After correction, several between-group differences remained significant: white-cell count (WBC; Cohen’s d = 0.90; FDR-*p* = 0.001), neutrophil count (rank-biserial |r| = 0.46; FDR-*p* = 0.002), platelet count (d = 0.73; FDR-*p* = 0.006), haemoglobin (|r| = 0.42; FDR-*p* = 0.005), haematocrit (|r| = 0.31; FDR-*p* = 0.037), mean corpuscular volume (MCV; |r| = 0.32; FDR-*p* = 0.031), fasting glucose (|r| = 0.55; FDR-*p* < 0.001), CRP (|r| = 0.57; FDR-*p* = 0.021) and HbA1c (|r| = 0.62; FDR-*p* = 0.021).

By contrast, lymphocyte count did not retain significance after FDR adjustment (Cohen’s d = 0.47; FDR-*p* = 0.076). Hepatic and renal parameters (aspartate aminotransferase (AST), alanine aminotransferase (ALT), alkaline phosphatase (ALP), γ-glutamyl transferase (GGT), total and direct bilirubin, urea, creatinine) did not differ between groups before or after correction. The CRP and HbA1c comparisons should be interpreted cautiously owing to substantial missingness in controls (CRP 11/36; HbA1c 7/36) and are therefore considered exploratory. Unchanged hepatic and renal biochemical markers (AST, ALT, ALP, GGT, bilirubin, urea, creatinine) indicate that, in this predominantly young-to-middle-aged surgical cohort, overt laboratory evidence of end-organ hepatic or renal injury was not yet manifest, despite a clear systemic inflammatory–metabolic phenotype. Normal-group average liver and kidney tests therefore support the biological specificity of the inflammatory differences rather than global multi-organ laboratory derangement.

### 2.7. Sensitivity Analyses

Because several design and analytical issues could affect inference, sensitivity analyses were performed. First, repeating the primary Mann–Whitney comparison after excluding the 15 out-of-range TRPA1 values (>900 pg/mL; remaining *n* = 36/27) did not alter the conclusion (median 277.1 vs. 290.3 pg/mL; *p* = 0.744; Hodges–Lehmann −7.60 pg/mL, 95% CI −60.10 to 47.11; age-/sex-adjusted OR for log-TRPA1, *p* = 0.986). Second, excluding the three obesity arm participants with BMI 39.06 kg/m^2^ (remaining *n* = 39/36) likewise left the primary null finding unchanged (*p* = 0.393; Hodges–Lehmann −28.30 pg/mL, 95% CI −192.26 to 53.90; adjusted log-TRPA1, *p* = 0.820). Third, restricting controls to a stricter definition (BMI < 25 kg/m^2^ and no recorded dyslipidaemia; *n* = 42/21) again did not yield a significant TRPA1 difference (*p* = 0.290; Hodges–Lehmann −34.00 pg/mL, 95% CI −300.45 to 41.23; adjusted log-TRPA1, *p* = 0.454). Fourth, age- and sex-adjusted logistic models using continuous log-TRPA1 (primary) and the exploratory dichotomised exposure produced concordant null inferences for TRPA1. Collectively, these checks suggest that the negative circulating result is not driven solely by out-of-range extrapolations, borderline BMI values, or the most liberal control definition; however, because the study was powered for a large effect, smaller differences remain possible.

## 3. Discussion

To our knowledge, this is the first prospective clinical study to measure circulating serum TRPA1 protein in people with class III obesity undergoing bariatric surgery and to compare it with contemporaneous controls. The pre-specified primary finding was that preoperative serum TRPA1 did not differ between groups (Hodges–Lehmann median difference −30.80 pg/mL, bootstrap 95% CI −227.43 to 43.02) and showed no independent association with obesity status after adjustment for age and sex when modelled continuously. Exploratory ROC analysis likewise showed no useful discriminatory capacity (AUC = 0.435, 95% CI 0.305–0.562). In this setting, serum TRPA1 is not supported as a stand-alone circulating biomarker; we deliberately avoid stronger language such as “ruling out its utility”, because the present evidence is limited to a single preoperative ELISA measurement in a modestly sized, demographically imbalanced cohort.

These findings are at first glance counterintuitive given the experimental rationale. In rodent and cellular models, TRPA1 participates in adipose energy balance regulation and can be engaged by obesity-associated endogenous agonists such as 4-HNE and hydrogen peroxide [11,17]; dietary TRPA1 agonists such as cinnamaldehyde have been linked to reduced visceral fat and thermogenic responses [14,15,17], and multi-agonist TRP strategies can improve glucose utilisation phenotypes in high-fat-diet models [16]. TRPA1 has also been discussed as a therapeutic target in metabolic disorders [12] and as an amplifier of inflammatory cascades [9,13]. Critically, nearly all of this literature concerns tissue expression, sensory–autonomic reflexes, or functional channel activation—not validated serum protein quantification.

Separating tissue-level TRPA1 biology from circulating TRPA1 is therefore central to interpreting the negative primary result. As a membrane-bound ion channel, TRPA1’s serum immunoreactivity likely represents ectodomain shedding, vesicular release, and/or cellular debris rather than electrophysiological channel activity [5,9,20]. A negative serum comparison does not refute adipose or immune cell TRPA1 involvement; rather, it suggests a potential translational disconnect between the extensive tissue-level evidence base and circulating TRPA1 as a biomarker. Sparse human ELISA data in other diseases [22] and blood-based epigenetic or leukocyte studies [21,23] further underscore that circulating read-outs remain methodologically immature relative to tissue biology. Several additional explanations may account for the null circulating finding. The groups were imbalanced for age and sex; although accounted for in the adjusted continuous model—where TRPA1 remained non-significant—residual structural confounding may persist. Broad within-group variability and upper-end outliers (Figure 1), including values beyond the nominal ELISA range, reduced precision. The a priori sample-size calculation targeted a large effect (Cohen’s d = 0.8); accordingly, the present result is best summarised as no evidence of a large between-group difference, not as proof that no smaller difference exists. Importantly, the null TRPA1 result must be read against a robust inflammatory–metabolic phenotype: after FDR correction, people with obesity had significantly higher WBC, neutrophils, platelets, fasting glucose, CRP, and HbA1c and lower haemoglobin, haematocrit, and MCV—consistent with chronic low-grade inflammation—whereas liver enzymes, bilirubin, urea and creatinine were unchanged. These secondary findings validate the cohort biologically while reinforcing that ELISA-measured serum TRPA1 did not track established inflammatory surrogates in this dataset.

Regarding clinical relevance, participants already met anthropometric criteria for class III obesity and were scheduled for sleeve gastrectomy; there is therefore no clinical need to “re-diagnose” obesity with a serum assay. Our objective was narrower: to test whether circulating TRPA1 carries incremental preoperative biological information. On that specific question, the present data are negative. Candidate markers for future multivariable panels—based on signals that did differ in this cohort—include CRP, leukocyte/neutrophil counts, platelet count, fasting glucose, and HbA1c, always interpreted alongside age and sex. Whether tissue TRPA1 (mRNA, protein localisation, or functional calcium responses) adds value beyond these conventional markers remains an open experimental question. The weak negative correlation between TRPA1 and HbA1c in the obesity group (rs = −0.314; *p* = 0.043; *n* = 42; Table S2) is of uncertain significance. Directionally, it runs counter to a simple inflammatory activation hypothesis, was not pre-specified, was not seen in controls, and does not survive multiplicity adjustment; it should be regarded as hypothesis-generating only.

Crucially, the absence of a between-group serum difference does not exclude a role for TRPA1 in tissue-level inflammation, since serum levels may not capture receptor density, activation state, or downstream calcium signalling in adipose, immune, or gastrointestinal tissue [5,6,9,11,13].

### Limitations

Several limitations should be acknowledged. First, the cross-sectional, preoperative-only design precludes assessment of longitudinal TRPA1 dynamics after metabolic remodelling induced by sleeve gastrectomy. Second, the age and sex imbalance, though statistically adjusted, is a structural design limitation. The control group was not restricted to normal body mass index (13 of 36 controls had a BMI ≥ 25 kg/m^2^), and three participants in the obesity arm had a BMI of 39.06 kg/m^2^, just below the class III threshold of 40 kg/m^2^; both features narrow the intended anthropometric contrast (sensitivity analyses are reported in Section 2.7). Third, circulating serum TRPA1 is an imperfect proxy for tissue-level channel expression or electrophysiological activity. Follow-up work using adipose biopsies with RT-PCR and immunohistochemistry could clarify expression and localisation but would still not establish channel function; functional assays (for example, agonist-evoked calcium imaging or electrophysiology in relevant cells) would be required to assess activity. Fourth, CRP, ALP and HbA1c had substantial missingness (CRP 19/42 versus 11/36; ALP 18/42 versus 21/36; HbA1c 42/42 versus 7/36). Missingness was not random by design: CRP and HbA1c were not part of the mandatory preoperative panel for all controls attending routine screening and were therefore available only when clinically requested or when residual serum volume permitted add-on testing; ALP missingness reflected intermittent reagent/panel availability in the hospital laboratory during the recruitment window. Analyses used available cases without imputation. Fifth, dietary intake, physical activity, and smoking were not systematically quantified. Available comorbidity fields showed hypertension in 4/42 and type 2 diabetes in 3/42 participants in the obesity arm (plus isolated CAD/COPD/asthma/PHT codes), whereas no hypertension or OSAS was recorded among controls; nevertheless, dyslipidaemia was recorded in 4/36 controls and other comorbidity flags in 2/36, inconsistent with the strictest metabolically healthy definition—hence the stricter control sensitivity analysis. Sixth, although the cohort exceeded the a priori sample-size target, that target assumed a large effect (Cohen’s d = 0.8); with *n* = 42/36, the post hoc minimum detectable effect at 80% power and α = 0.05 (two-tailed) is approximately d ≈ 0.65, so smaller but potentially meaningful differences in serum TRPA1 cannot be excluded. Finally, TRPA1 was quantified with a commercial ELISA as single-well measurements without in-study spike recovery or dilutional linearity; fifteen values exceeded the kit’s stated analytical range (3–900 pg/mL) and were not diluted/re-assayed; samples were stored at −20 °C for approximately three months, and performance characteristics remain manufacturer-stated. Independent assay validation and paired tissue measurements would strengthen confidence in the circulating signal [20].

## 4. Materials and Methods

### 4.1. Study Design and Ethical Approval

This single-centre, prospective, controlled observational study was conducted at the Department of General Surgery and the Biochemistry Laboratory of Elazığ Fethi Sekin City Hospital, Elazığ, Türkiye. It was approved by the Non-Interventional Research Ethics Committee (approval no. 2025/20-24; date of approval: 4 December 2025) and conducted in accordance with the 2013 Declaration of Helsinki. Written informed consent was obtained from all participants before enrolment [24]. Reporting follows STROBE guidelines [25].

### 4.2. Participants

The study group comprised 42 consecutive adults with class III obesity (BMI ≥ 40 kg/m^2^) scheduled for laparoscopic sleeve gastrectomy at the study centre. Three enrolled surgical candidates had a measured BMI of 39.06 kg/m^2^ at the research anthropometry visit (rounded clinical BMI had been recorded as 39–40 kg/m^2^ during surgical workup); these participants were retained in the primary analysis and excluded in a sensitivity analysis (Section 2.7). The control group comprised 36 consecutively recruited adults without class III obesity attending routine health screening during the same period, intended to have no known metabolic, inflammatory, or chronic systemic disease and not to be receiving anti-inflammatory or glucose-lowering therapy. As detailed in Limitations, four controls had recorded dyslipidaemia and 13 controls had BMI ≥ 25 kg/m^2^; these departures from the strictest control definition are acknowledged and examined in sensitivity analyses. We therefore refer to this arm as the control group rather than uniformly as “healthy controls”.

Inclusion criteria (obesity group) were age ≥ 18 years; class III obesity confirmed by standard anthropometric measurement; scheduled laparoscopic sleeve gastrectomy; preoperative biochemical and haematological data in the hospital information management system (HIMS); and signed informed consent. Controls required age ≥ 18 years; absence of obesity; attendance for routine screening without acute or chronic illness; and signed informed consent.

Exclusion criteria for both groups were age < 18 years, active malignancy, rheumatologic or autoimmune disease, acute infectious or systemic inflammatory disease, pregnancy or lactation, and refusal or inability to provide written informed consent. For the obesity group, patients undergoing a bariatric procedure other than laparoscopic sleeve gastrectomy were also excluded.

Missing data: variables with substantial missingness are identified in Table 3 (marked §). CRP was available in 19/42 people with obesity and 11/36 controls, ALP in 18/42 and 21/36, and HbA1c in all 42 people with obesity but only 7/36 controls. These variables are reported descriptively and in exploratory analyses only. Spearman correlation pair numbers are reported in Table S2; insulin and HOMA-IR were not analysable in controls (*n* < 5). Participants were not excluded solely because of missing laboratory measurements. Analyses were performed using available-case analysis for each variable, without imputation. Therefore, the number of participants included in individual analyses varied according to data availability and is reported for each variable in Table 3 and Table S2.

### 4.3. Blood Sampling and TRPA1 Measurement

Peripheral venous blood (5 mL) was collected from each participant by standard antecubital venipuncture between 08:00 and 10:00 a.m. after an overnight fast, in addition to routine preoperative sampling. Samples were allowed to clot for 10 min and were then centrifuged at 3000 rpm (approximately 1500× *g*) for 10 min; serum aliquots were stored at −20 °C for approximately 3 months until batch analysis and underwent a single freeze–thaw cycle. Serum TRPA1 concentrations were measured using a commercial human TRPA1 ELISA kit (Shanghai YL Biotech Co., Ltd., Shanghai, China; catalogue no. YLA4127HU; analytical range, 3–900 pg/mL; sensitivity, 1.48 pg/mL; manufacturer-reported intra- and inter-assay coefficients of variation, <8% and <10%, respectively), according to the manufacturer’s instructions.

A series of standard dilutions was prepared by serially diluting the supplied 960 pg/mL standard. Each well, except the blank well, received 50 µL of streptavidin–horseradish peroxidase. For the standard wells, 50 µL of the appropriate standard dilution was added, whereas the sample wells received 40 µL of serum and 10 µL of TRPA1 antibody. The plate was sealed and incubated at 37 °C for 1 h. Following incubation, the wells were washed five times with a 25-fold diluted washing solution using a Combiwash microplate washer (HUMAN Gesellschaft für Biochemica und Diagnostica mbH, Wiesbaden, Germany). Subsequently, 50 µL each of chromogen solutions A and B were added to all wells, followed by incubation at 37 °C for 10 min. The reaction was terminated by adding 50 µL of stop solution, and absorbance was measured at 450 nm within 15 min using a ChroMate 4300 microplate reader (Awareness Technology, Inc., Palm City, FL, USA). Sample concentrations were calculated using a linear calibration curve. All samples were analysed once in single wells by the same experienced laboratory specialist, who was blinded to group allocation, and the results were expressed in pg/mL. No in-study spike recovery or dilution linearity experiments were performed. Fifteen measurements exceeded the kit’s stated upper analytical limit of 900 pg/mL; these values were retained in the primary analysis as reported by the plate reader calibration curve and were excluded from the sensitivity analysis because the samples had not been diluted and re-assayed.

### 4.4. Secondary Laboratory Measurements

Complete blood count parameters (WBC, haemoglobin, haematocrit, MCV, platelet count, neutrophils, lymphocytes) were measured on a Beckman Coulter DxH 800 haematology analyser (Beckman Coulter, Inc., Brea, CA, USA); serum biochemistry (glucose, urea, creatinine, AST, ALT, ALP, GGT, total and direct bilirubin) was measured on a Beckman Coulter AU5800 analyser (Beckman Coulter, Inc., Brea, CA, USA); and insulin was measured by Beckman Coulter UniCel DxI 800 chemiluminescence immunoassay (Beckman Coulter, Inc., Brea, CA, USA). Insulin resistance was estimated as HOMA-IR = [fasting glucose (mmol/L) × fasting insulin (µU/mL)]/22.5. HbA1c was measured by high-performance liquid chromatography on a Premier Hb9210 analyser (Trinity Biotech plc, Bray, Ireland) and CRP by immunoturbidimetry on the AU5800 platform (Beckman Coulter, Inc., Brea, CA, USA).

### 4.5. Statistical Analysis

Serum TRPA1 and haematological, metabolic, and biochemical parameters were compared between people with class III obesity and controls. Analyses were performed in Python 3.11 (Python Software Foundation, Wilmington, DE, USA) using SciPy 1.13 (Enthought, Austin, TX, USA), Statsmodels 0.14 and scikit-learn 1.5 (https://scipy.org; https://www.statsmodels.org; https://scikit-learn.org; accessed on 15 March 2026) and cross-verified in IBM SPSS Statistics 26 (IBM Corp., Armonk, NY, USA). Normality was tested by the Shapiro–Wilk test (*n* ≤ 50). Normally distributed variables are presented as mean ± SD and compared by the independent-samples *t*-test, with Cohen’s d as the effect size [26]; non-normally distributed variables are presented as median (IQR Q1–Q3) and compared by the Mann–Whitney U test, with rank-biserial correlation (r). For *t*-tests, 95% confidence intervals are mean difference ± 1.96·SE; for Mann–Whitney comparisons, the Hodges–Lehmann median difference with bootstrap 95% CI (2000 resamples, seed 42) is reported and is presented for the primary TRPA1 contrast. Secondary outcomes were adjusted for multiplicity by the Benjamini–Hochberg FDR procedure, and only FDR-corrected *p*-values are used to claim significance for them [27]; the primary TRPA1 comparison was not adjusted. Associations between preoperative TRPA1 and metabolic parameters were examined by Spearman rank correlation (rs) in both groups, with bootstrap 95% CI (Fisher-z transformation) and exact pair numbers reported in Table S2. The primary adjusted analysis modelled continuous log-transformed TRPA1 together with age and sex in binary logistic regression (likelihood-ratio test and Nagelkerke R^2^). Dichotomisation at the Youden index ROC threshold was retained only as an exploratory analysis. Discriminatory capacity was evaluated by ROC analysis with class III obesity as the positive class, computing the area under the curve (AUC) with a bootstrap 95% CI (2000 resamples), the Youden-index-derived optimal threshold, and the corresponding sensitivity and specificity (Table S1) [28]. Sensitivity analyses excluded out-of-range TRPA1 values, excluded BMI 39.06 kg/m^2^ participants, and restricted controls to BMI < 25 kg/m^2^ without recorded dyslipidaemia. Two-tailed *p* < 0.05 was considered statistically significant.

Sample size: a priori power analysis in G*Power 3.1.9.7 (Heinrich-Heine-Universität Düsseldorf, Düsseldorf, Germany; https://www.psychologie.hhu.de/arbeitsgruppen/allgemeine-psychologie-und-arbeitspsychologie/gpower; accessed on 10 November 2025) indicated that 35 participants per group were required for 80% power at α = 0.05 to detect a large effect (Cohen’s d = 0.8); the final cohort (42 with obesity, 36 controls) met or exceeded this threshold. The study was therefore powered only for a large effect, and non-significant results should not be interpreted as evidence of equivalence.

## 5. Conclusions

In this prospective, controlled study of bariatric surgery candidates, preoperative serum TRPA1 did not distinguish people with class III obesity from contemporaneous controls. Median TRPA1 was marginally lower in the obesity group (309.8 vs. 340.5 pg/mL; *p* = 0.323), with a Hodges–Lehmann median difference of −30.80 pg/mL (bootstrap 95% CI −227.43 to 43.02). Continuous log-TRPA1 was not independently associated with group membership after adjustment for age and sex (OR per log-unit 0.88, 95% CI 0.46–1.69; *p* = 0.699); an exploratory dichotomised model similarly showed no independent TRPA1 effect. ROC analysis confirmed an absence of clinically useful discrimination (AUC 0.435, 95% CI 0.305–0.562). These results provide no evidence of a large circulating difference—not proof that no smaller difference exists.

Against this negative primary circulating signal, the conventional inflammatory and metabolic profile behaved as expected: after false-discovery-rate correction, the cohort with obesity showed significantly higher leukocyte, neutrophil, and platelet counts together with lower haemoglobin and higher fasting glucose, C-reactive protein and HbA1c, reaffirming the low-grade inflammatory state of severe obesity. Serum TRPA1 did not track these markers. Circulating immunoreactive TRPA1 may be an imperfect surrogate for membrane-bound, tissue-level channel expression or activity, so a biologically relevant tissue role cannot be excluded from a single peripheral measurement. Translationally, these findings do not support preoperative serum TRPA1 as a stand-alone risk stratification biomarker in bariatric surgery candidates; future strategies will more plausibly rest on multivariable panels integrating demographic and established inflammatory covariates, with tissue-based and functional TRPA1 assays reserved for mechanistic follow-up. Until such data are available, circulating TRPA1 remains a mechanistically interesting but clinically unproven analyte in severe obesity.

## Figures and Tables

**Figure 1:**
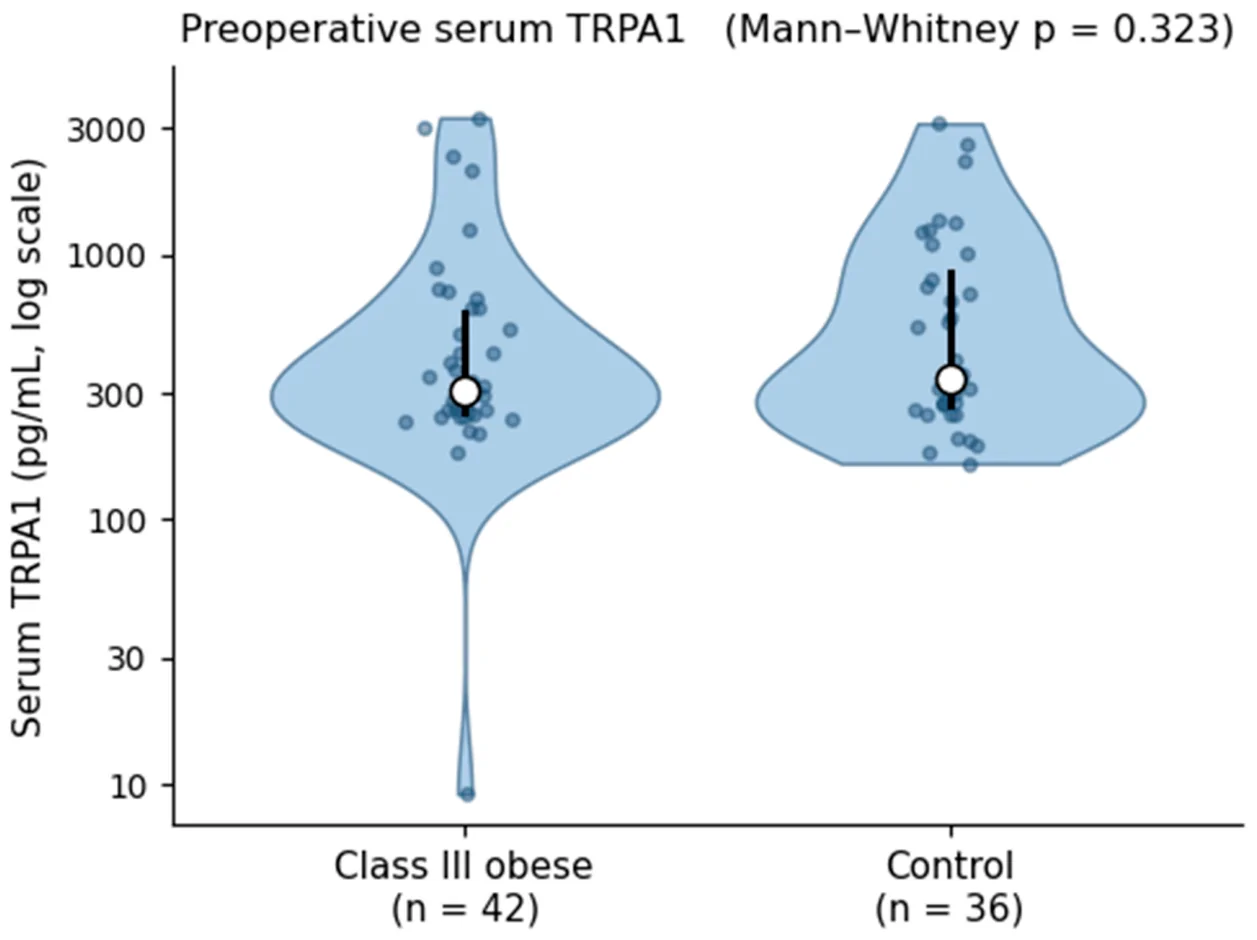
Violin plot of preoperative serum Transient Receptor Potential Ankyrin 1 (TRPA1) concentrations in people with class III obesity (*n* = 42) and control participants (*n* = 36) on a log-scaled *y*-axis. Overlaid central summary markers indicate median and interquartile range. Distributions are right-skewed with substantial overlap and upper-end outliers (Mann–Whitney U: *p* = 0.323; rank-biserial r = 0.13).

**Figure 2:**
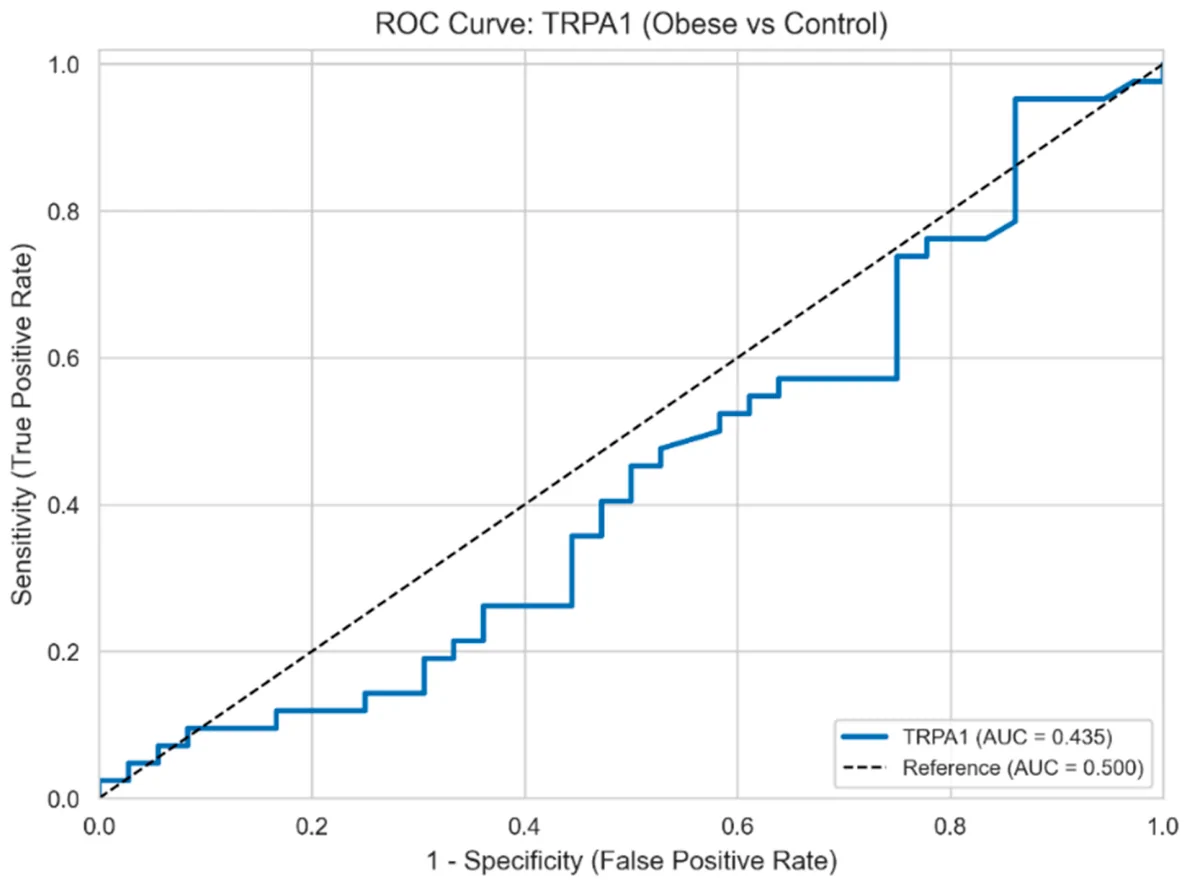
Receiver operating characteristic (ROC) curve of preoperative serum Transient Receptor Potential Ankyrin 1 (TRPA1) for discrimination between participants with class III obesity and control participants (area under the curve 0.435; 95% CI 0.305–0.562). The Youden-optimal threshold was 524 pg/mL (sensitivity 73.8%; specificity 44.4%; Table S1).

**Figure 3:**
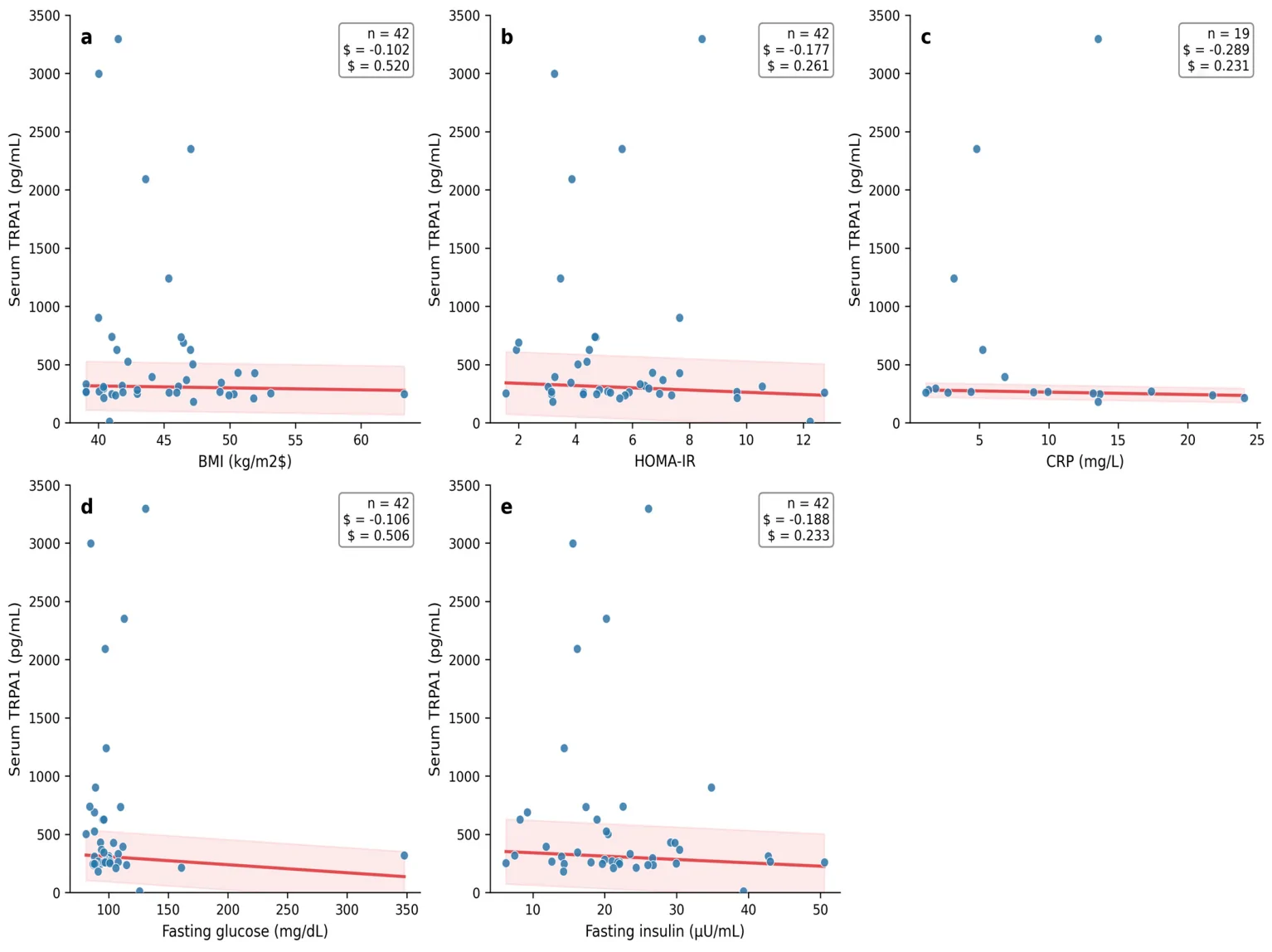
Correlation analyses between preoperative serum Transient Receptor Potential Ankyrin 1 (TRPA1) and metabolic-inflammatory parameters in the class III obesity cohort based on individual-participant data. (**a**) TRPA1 versus body mass index; (**b**) TRPA1 versus HOMA-IR; (**c**) TRPA1 versus C-reactive protein; (**d**) TRPA1 versus fasting glucose; (**e**) TRPA1 versus fasting insulin. Panel letters are placed inside each panel to avoid overlap with axis titles. Points are individual participants; the red line is a Theil–Sen robust trend with an approximate variability band for visualisation. Spearman coefficients (rs) are shown in each panel; all associations were weak and non-significant. *n* = 42 for all panels except (**c**), where *n* = 19 owing to missing C-reactive protein measurements. White-cell count and HbA1c correlations, and the corresponding control-group analyses, are reported in Table S2. HOMA-IR, homeostasis model assessment of insulin resistance.

**Table 1:** Demographic and anthropometric characteristics of the study groups.

Variable	Class III Obesity (*n* = 42)	Control (*n* = 36)	*p*-Value	ES
Age (years)	36.00 (25.50–44.00)	24.50 (20.75–31.25)	0.0002 ‡	r = −0.49
Height (cm)	165.00 (160.0–169.75)	174.00 (169.75–177.25)	<0.001 ‡	r = 0.59
Weight (kg)	120.00 (110.0–138.25)	71.00 (66.50–80.00)	<0.001 ‡	r = −0.97
BMI (kg/m^2^)	43.84 (41.02–47.15)	24.14 (22.24–26.29)	<0.001 ‡	r = −1.00
Sex, female/male	31/11 (74%/26%)	12/24 (33%/67%)	<0.001 †	φ = 0.42
Surgery type	Sleeve gastrectomy (100%)	—	—	—
Hospital stay (days)	6.00 (5.00–7.00)	—	—	—

Data are median (interquartile range) or *n* (%). † Chi-square test; ‡ Mann–Whitney U test. BMI, body mass index; ES, effect size (rank-biserial r or φ).

**Table 2:** Primary confounder-adjusted binary logistic regression: continuous log-transformed serum TRPA1 as a predictor of obesity status (adjusted for age and sex).

Variable	β Coefficient	SE	*p*-Value	95% CI
log(serum TRPA1)	−0.1285	0.3327	0.699	−0.7805 to 0.5235
Age (years)	0.0964	0.0311	0.002	0.0354 to 0.1573
Sex (male = 1)	−1.5465	0.5456	0.005	−2.6159 to −0.4772

Primary adjusted model with continuous natural-log TRPA1 (*n* = 78). Model fit: LR χ^2^ = 26.2, *p* < 0.001; Nagelkerke R^2^ = 0.38. Odds ratio for log-TRPA1 = 0.88 (95% CI 0.46–1.69). The exploratory dichotomised model (low TRPA1 ≤ 524 pg/mL: OR 2.30, 95% CI 0.75–7.04; *p* = 0.146) is not interpreted as a validated clinical cut-off. CI, confidence interval; SE, standard error; TRPA1, Transient Receptor Potential Ankyrin 1.

**Table 3:** Serum TRPA1, haematological and biochemical comparisons between groups.

Parameter (Unit)	Class III Obesity	Control	*p* (Raw)	*p* (FDR) †	ES ‡	*n* (O/C)
Serum TRPA1 (pg/mL) [PRIMARY]	309.75 (254.15–600.35)	340.50 (268.93–856.32)	0.323	—	r = 0.13	42/36
WBC (10^3^/µL)	9.00 ± 2.02	7.37 ± 1.60	0.0002	**0.0009**	d = 0.90	42/35
Neutrophil (10^3^/µL)	5.47 (4.42–7.02)	4.24 (3.46–5.06)	0.001	**0.0016**	r = −0.46	42/35
Lymphocyte (10^3^/µL)	2.41 ± 0.73	2.13 ± 0.48	0.048	0.076	d = 0.47	42/35
Platelet (10^3^/µL)	323.76 ± 78.52	271.80 ± 64.09	0.002	**0.006**	d = 0.73	42/35
Haemoglobin (g/dL)	13.65 (13.12–15.03)	15.50 (13.65–16.65)	0.002	**0.005**	r = 0.42	42/35
Haematocrit (%)	41.80 (39.65–44.90)	45.70 (40.05–48.85)	0.022	**0.037**	r = 0.31	42/35
MCV (fL)	82.10 (78.15–88.00)	87.10 (84.60–88.70)	0.017	**0.031**	r = 0.32	42/35
Glucose (mg/dL)	97.00 (89.50–105.50)	87.50 (83.00–91.00)	<0.001	**0.0002**	r = −0.55	42/34
Insulin (µU/mL)	20.71 (15.71–26.48)	—	—	—	—	42/—
HOMA-IR	4.98 (3.85–6.88)	—	—	—	—	42/—
HbA1c (%)	5.80 (5.53–6.00)	5.20 (4.95–5.35)	0.010	**0.021**	r = −0.62	42/7 §
CRP (mg/L)	8.91 (3.78–13.61)	1.89 (1.30–3.15)	0.010	**0.021**	r = −0.57	19 §/11 §
AST (U/L)	21.00 (18.00–27.00)	22.50 (18.25–24.75)	0.842	0.883	r = −0.03	42/34
ALT (U/L)	25.00 (17.25–38.00)	24.00 (14.50–32.00)	0.590	0.683	r = −0.07	42/34
ALP (U/L)	83.00 (76.50–101.25)	78.00 (68.00–84.00)	0.205	0.281	r = −0.24	18 §/21 §
GGT (U/L)	23.00 (17.00–33.00)	20.00 (12.75–30.25)	0.135	0.198	r = −0.22	29 §/32 §
Total bilirubin (mg/dL)	0.57 (0.43–0.72)	0.57 (0.42–0.85)	0.956	0.956	r = −0.01	38 §/21 §
Direct bilirubin (mg/dL)	0.13 (0.08–0.16)	0.11 (0.08–0.19)	0.830	0.883	r = 0.04	38 §/21 §
Urea (mg/dL)	25.50 (21.25–28.75)	23.00 (22.00–27.00)	0.454	0.555	r = −0.10	42/34
Creatinine (mg/dL)	0.68 ± 0.14	0.71 ± 0.17	0.396	0.512	d = −0.20	42/34

Data are mean ± SD or median (IQR). Normally distributed variables were compared by independent *t*-test (effect size: Cohen’s d); non-normal variables by Mann–Whitney U (effect size: rank-biserial r). † FDR-corrected *p*-values (Benjamini–Hochberg) applied to secondary outcomes only; the primary outcome (TRPA1) is not subject to multiplicity correction and is reported raw. ‡ ES = effect size. § Indicates substantial missingness; interpret with caution. O = obesity group; C = control group. Bold FDR-*p* values are statistically significant after correction. ALP, alkaline phosphatase; ALT, alanine aminotransferase; AST, aspartate aminotransferase; CRP, C-reactive protein; FDR, false discovery rate; GGT, γ-glutamyl transferase; HbA1c, glycated haemoglobin; HOMA-IR, homeostasis model assessment of insulin resistance; IQR, interquartile range; MCV, mean corpuscular volume; SD, standard deviation; TRPA1, Transient Receptor Potential Ankyrin 1; WBC, white-cell count.

## Data Availability

The data presented in this study are available on request from the corresponding author due to privacy and ethical restrictions. Anonymised individual participant data (excluding identifiable variables) will be shared upon reasonable request, subject to approval by the institutional ethics committee.

## Supplementary Materials

The following supporting information can be downloaded at: https://www.mdpi.com/article/10.3390/ijms27198919/s1.

## Abbreviations

The following abbreviations are used in this manuscript:

4-HNE	4-hydroxynonenal
ALP	alkaline phosphatase
ALT	alanine aminotransferase
AST	aspartate aminotransferase
AUC	area under the curve
BMI	body mass index
CI	confidence interval
CRP	C-reactive protein
ELISA	enzyme-linked immunosorbent assay
FDR	false discovery rate
GGT	γ-glutamyl transferase
HbA1c	glycated haemoglobin
HOMA-IR	homeostasis model assessment of insulin resistance
IQR	interquartile range
MCV	mean corpuscular volume
MDA	malondialdehyde
OR	odds ratio
ROC	receiver operating characteristic
ROS	reactive oxygen species
SD	standard deviation
STROBE	Strengthening the Reporting of Observational Studies in Epidemiology
TRPA1	Transient Receptor Potential Ankyrin 1
WBC	white-cell count
