# Supplementary material for: Preoperative Serum Transient Receptor Potential Ankyrin 1 (TRPA1) Levels in Class III Obesity: A Prospective Controlled Observational Study
Source: Int J Mol Sci. 2026 Oct 8;27(19):8919. doi: 10.3390/ijms27198919 (PMC13665438; doi:10.3390/ijms27198919)
Supplement: Supplementary file 1 [file ijms-27-08919-s001.zip › ijms-4561556-supplementary.pdf]

Supplementary Materials

Preoperative Serum Transient Receptor Potential Ankyrin 1 (TRPA1) Levels in Class III Obesity: A Prospective Controlled Observational Study

Nizamettin Kutluer, Onur Ağ, Tamer Gündoğdu, Mehmet Zeki Öğüt, Denizhan Kılıç, Hakan Ayyıldız, Serhat Doğan and Mehmet Buğra Bozan

Table S1. Diagnostic performance of preoperative serum TRPA1 for discriminating participants with class III obesity from control participants (ROC analysis).

Parameter	Value
Area under the curve (AUC)	0.435 (95% CI 0.305–0.562)
Optimal cut-off (Youden index)	524 pg/mL
Sensitivity	73.8%
Specificity	44.4%
Youden index (J)	0.183
Discriminatory performance	Poor — no clinically useful discrimination

ROC analysis with class III obesity as the positive class; an AUC < 0.50 indicates that higher serum TRPA1 was associated with control rather than obesity status. The 95% confidence interval was estimated by bootstrap (2000 resamples). AUC, area under the curve; CI, confidence interval; ROC, receiver operating characteristic; TRPA1, Transient Receptor Potential Ankyrin 1.

Table S2. Spearman correlations between preoperative serum TRPA1 and metabolic parameters in the class III obesity and control groups.

Variable	Obesity rs (p)	Control rs (p)	n (obesity)	n (control)
BMI (kg/m^2)	−0.102 (0.520)	−0.140 (0.416)	42	36
HOMA-IR	−0.177 (0.261)	NA *	42	<5
Fasting glucose (mg/dL)	−0.106 (0.506)	−0.015 (0.933)	42	34
Fasting insulin (μU/mL)	−0.188 (0.233)	NA *	42	<5
CRP (mg/L)	−0.289 (0.231)	−0.105 (0.745)	19	12
WBC (10^3/μL)	−0.129 (0.417)	−0.231 (0.181)	42	35
HbA1c (%)	−0.314 (0.043)	+0.198 (0.670)	42	7

rs = Spearman's rank correlation coefficient; all p-values are two-tailed. * NA = not analysable in controls (n < 5 owing to missing insulin/HOMA-IR data). CRP and HbA1c control correlations are limited by missing data (n = 12 and n = 7, respectively) and should be interpreted with caution. BMI, body mass index; CRP, C-reactive protein; HbA1c, glycated haemoglobin; HOMA-IR, homeostasis model assessment of insulin resistance; TRPA1, Transient Receptor Potential Ankyrin 1; WBC, white-cell count.
